# Differential impact of severe drought on infant mortality in two sympatric neotropical primates

**DOI:** 10.1098/rsos.200302

**Published:** 2020-04-01

**Authors:** Fernando A. Campos, Urs Kalbitzer, Amanda D. Melin, Jeremy D. Hogan, Saul E. Cheves, Evin Murillo-Chacon, Adrián Guadamuz, Monica S. Myers, Colleen M. Schaffner, Katharine M. Jack, Filippo Aureli, Linda M. Fedigan

**Affiliations:** 1 Department of Anthropology, University of Texas at San Antonio, San Antonio, TX, USA; 2 Department of Biology, McGill University, Montreal, Quebec, Canada; 3 Department of Anthropology and Archaeology, University of Calgary, Calgary, AB, Canada; 4 Alberta Children's Hospital Research Institute, University of Calgary, Calgary, AB, Canada; 5 Área de Conservación Guanacaste, Guanacaste, Costa Rica; 6 Department of Psychology, Adams State University, Alamosa, CO, USA; 7 Department of Anthropology, Tulane University, New Orleans, LA, USA; 8 Instituto de Neuroetología, Universidad Veracruzana, Xalapa, Mexico; 9 Research Centre in Evolutionary Anthropology and Palaeoecology, Liverpool John Moores University, Liverpool, UK

**Keywords:** climate change, tropical dry forest, *Cebus capucinus imitator*, *Ateles geoffroyi*, Costa Rica, demography

## Abstract

Extreme climate events can have important consequences for the dynamics of natural populations, and severe droughts are predicted to become more common and intense due to climate change. We analysed infant mortality in relation to drought in two primate species (white-faced capuchins, *Cebus capucinus imitator,* and Geoffroy's spider monkeys, *Ateles geoffroyi*) in a tropical dry forest in northwestern Costa Rica. Our survival analyses combine several rare and valuable long-term datasets, including long-term primate life-history, landscape-scale fruit abundance, food-tree mortality, and climate conditions. Infant capuchins showed a threshold mortality response to drought, with exceptionally high mortality during a period of intense drought, but not during periods of moderate water shortage. By contrast, spider monkey females stopped reproducing during severe drought, and the mortality of infant spider monkeys peaked later during a period of low fruit abundance and high food-tree mortality linked to the drought. These divergent patterns implicate differing physiology, behaviour or associated factors in shaping species-specific drought responses. Our findings link predictions about the Earth's changing climate to environmental influences on primate mortality risk and thereby improve our understanding of how the increasing severity and frequency of droughts will affect the dynamics and conservation of wild primates.

## 1. Background

There is growing recognition that the Earth's changing climate is strongly affecting tropical ecosystems around the world [1,2]. Many tropical plant and animal species have evolved narrow environmental tolerances, making them highly vulnerable to even small changes in climate conditions [3,4]. Although there is considerable uncertainty surrounding the relationship between extreme climate events and global climate change [5,6], there is growing evidence of potentially catastrophic ecological dangers posed by the interaction of the ‘press’ of gradual climate change with increasingly severe ‘pulses’ of extreme events [7]. Recent research has brought attention to the importance of extreme climate events for a wide range of organisms and ecological systems, and has revealed that some biological responses to environmental factors may be evident only when a certain critical threshold is exceeded [8–11]. Across taxa, extreme weather and climate events are commonly associated with negative responses, including population declines and local extirpation [12]. At a global scale, severe droughts are likely to become more common, more intense, or set in more quickly under climate change [13,14]. This confluence of factors demands concentrated research efforts to understand how the dynamics of natural populations are linked to climate variability.

Among mammals, primates are the order with greatest exposure to droughts and cyclones because many threatened primates occupy areas that are highly susceptible to these threats [15]. However, primate species differ in their susceptibility to these extreme events depending on behavioural, ecological, morphological and life-history traits [16]. In a wide range of natural primate populations, extremes in climatic water balance, including both severe drought and excessive rain, have been associated with elevated mortality [17–20], reduced fecundity [21–23], delayed birth seasons [22], declines in population density [24] and demographic perturbations [25]. Furthermore, extreme weather events can trigger periods of critically low resource availability [26]. For example, in vervet monkeys (*Chlorocebus pygerythrus*), high mortality during a severe drought was presaged by reduced food availability and an increase in physiological markers of stress [19]. In savannah baboons (*Papio* spp.), female infants that experienced drought conditions in early life had lower fertility as adults compared to other females, which suggests that experiencing the drought constrained their development and the costs were carried throughout life [27].

In spite of this growing literature, identifying effects of climate change and climate variability on primate populations remains challenging. First, primates have exceptionally slow life histories and low reproductive rates [28], which means that the necessary biographical data accumulate slowly and adequate sample sizes for robust inference are difficult to achieve. Second, most primates have high capacities to use behavioural responses to cope with changing and challenging environments [29], and this flexibility may make their demographic rates relatively insensitive to environmental conditions [30]. Life-history theory generally predicts that adult survival in long-lived, iteroparous organisms such as primates should be strongly buffered against fluctuating environmental conditions [31,32]. Strong environmental signals in the vital rates (i.e. survival and reproduction) of primates are therefore often absent, or at least difficult to detect, compared to species that are short-lived and reproduce quickly. But when such signals do exist, they are typically most evident in the survival or production of infants [33].

We investigated sources of mortality risk among infant primates in a seasonally dry tropical forest in northwestern Costa Rica, with particular focus on drought. Seasonally dry tropical forests throughout the world are highly sensitive to changes in rainfall regimes because water availability plays a critical role in determining the length and severity of wet and dry seasons as well as forest structure and function [34–36]. The effects of extreme climate states on fruiting and flowering in seasonally dry tropical forests therefore have the potential to cascade to many organisms that depend on the availability of these resources [37]. We combined rare and valuable long-term datasets, including phenological data on hundreds of fruit-producing trees, a daily record of local weather conditions, and decades of individual-based life-history data on two primate species: white-faced capuchins (*Cebus capucinus imitator*) and Geoffroy's spider monkeys (*Ateles geoffroyi*). These two primate populations occupy an area that experiences extreme seasonal and interannual variability in rainfall [38] and fruit availability [39,40]. Both species are primarily frugivorous, but the diet of capuchins also includes a substantial amount of arthropods, especially when fruit is less abundant [39,41,42]. Previous studies indicate that, in the capuchin population, infant survival rates (but not juvenile or adult female survival rates) vary in association with indices of the El Niño Southern Oscillation (ENSO), which are correlated with drought [25,33]. However, these population-level analyses were relatively coarse and did not account for other potentially important factors, such as resource abundance and infanticide by adult males.

We aimed to answer the following two questions: (i) Which environmental factors have the greatest impact on infant mortality in each species? (ii) Do infant mortality rates of our two study species show shared responses to similar environmental drivers? We expected infant mortality in both species to increase in association with drought, but we expected an association between infant mortality and fruit availability to be stronger in spider monkeys than in capuchins because the diet of spider monkeys is more specialized and reliant on ripe fruit [43]. Thus, fruit scarcity could lead to higher infant mortality as a result of reduced maternal energetic status or direct starvation among infants that have begun foraging independently. In addressing these questions, we seek to link predictions about the Earth's changing climate directly to environmental influences on mortality risk for each species to better understand the conservation implications of these predicted changes.

## 2. Methods

### 2.1. Study system

We carried out the research in Sector Santa Rosa (SSR) of the Área de Conservación Guanacaste, a UNESCO World Heritage Site in northwestern Costa Rica. The study area consists of tropical dry forest in different stages of regeneration following a long history of human disturbance [44]. Rainfall seasonality at SSR is exceptionally high, and interannual variability in rainfall is strongly influenced by the El Niño Southern Oscillation: drought periods coincide with strong El Niño episodes [25,38]. This occurred most recently in 2015, when SSR recorded 661 mm of annual rainfall, roughly 39% of the long-term annual mean (1708 mm) and less than 19% of the recorded maximum annual rainfall at this site (3498 mm). Three species of primates live in SSR: the white-faced capuchin, the mantled howler (*Alouatta palliata*), and the Geoffroy's spider monkey. The capuchin and spider monkey populations have been the subjects of intensive long-term field studies and are the focus of this paper. Both capuchins and spider monkeys give birth to single offspring, and throughout life, spider monkeys weigh 1.5–3 times as much as capuchins [45]. In white-faced capuchins, gestation is 5.5 months, the average interbirth interval is 26 months, and weaning age varies from 14 to 23 months [46–48]. In spider monkeys, gestation is 7.5 months, the average interbirth interval is 35 months, and weaning age varies from 19 to 31 months [49]. Male infanticide is a major source of infant mortality in the capuchin population at SSR [50–52], but it is not yet known whether male infanticide is a significant source of infant mortality in the spider monkey population. A comprehensive review of the natural history of the capuchin and spider monkey populations in SSR, including life-history traits, social structure and dynamics, and behavioural ecology, can be found in [53].

### 2.2. Primate life-history data

Intensive life-history data collection on multiple capuchin groups (seven groups total, two to five were under observation at any given time) began in 1986 and on a single spider monkey group in 2003, and continues to the present. The spider monkey group's home range overlaps greatly with the home ranges of the capuchin groups studied, and all individuals in these groups were individually recognized by experienced observers. The life-history data were collected by censusing each study group at monthly or sub-monthly intervals (near-daily during some periods), recording which individuals were present or absent. Following the approach detailed in [54], the census data were compiled into a biographical table that included date of birth (if known), sex, mother's identity (if known) and the way in which each animal entered and departed the study. Ways of entering the study included birth, immigration or being alive at the beginning of observation; ways of departing the study included death (confirmed or strongly suspected), emigration from a study group to a non-study group, permanent disappearance with unknown fate and still alive at end of observation (i.e. censored). We were interested in estimating infant survival, which we define as survival up to the minimum age of weaning in each species, which is 14 months for capuchins and 19 months for spider monkeys. All subjects included in the study were therefore unweaned infants. These ages are also lower than the age at natal dispersal in both species, therefore, we considered any infants that disappeared before these ages to have died. However, it is common not to observe some spider monkeys for extended periods due to their high degree of fission–fusion dynamics, which involves the splitting and merging of subgroups [55]. We treated cases of infants disappearing together with their mothers as censored at the time of last sighting, because they may or may not have died before reaching the minimum weaning age. We considered death to have occurred only in cases with confirmed or strong circumstantial evidence of death, including the disappearance of the infant but not the mother.

### 2.3. Climate data

Daily accumulated rainfall, minimum temperature and maximum temperature in SSR have been measured since July 1979 using standard rain gauges and thermometers located near the centre of the study area [38]. We used the R package SPEI v. 1.7 [56] to calculate a multi-scalar drought index, the Standardized Precipitation-Evapotranspiration Index (SPEI) [57], from the locally collected weather data, using the Hargreaves equation [58] to estimate evapotranspiration. The SPEI can be calculated over different time scales, reflecting differences among the Earth's ecosystems in rates of water accumulation, drought types and drought impacts. For example, the 12-month SPEI integrates climatic water balance over the previous 12 months. We calculated 1-month, 6-month and 12-month versions of the SPEI. Positive SPEI values represent water surplus (i.e. wet) conditions, while negative SPEI values represent water deficit (i.e. dry) conditions.

### 2.4. Available ripe fruit biomass

We calculated monthly estimates of available ripe fruit biomass at the landscape scale following methods described in detail in [59–61]. In brief, we used 11 years (2008–2018) of monthly phenological data on 46 fruit species to estimate temporal availability, and we used 13.94 ha of botanical transects arranged systematically over the study area to estimate density. See the electronic supplementary material for details. The 46 tree species represent at least 80% of the total diet for spider monkeys [62], and over 90% of the plant-based diet for capuchins [40]. Importantly, arthropods represent a relatively large component of the diet of capuchins (approx. 40% of gross energy intake), but not of spider monkeys [39]. We lack monthly data on arthropod abundance during the study period, but behavioural and nutritional data indicate that arthropod consumption by capuchins may be especially important during the early wet season months (June and July), when fruit abundance tends to be low [63]. The early wet season is also the only time of year when we have observed spider monkeys consuming animal matter (specifically, caterpillars).

### 2.5. Fruit tree mortality

We collected biographical data on fruit trees as part of the monthly phenological monitoring procedure used to estimate fruit biomass. Starting in February 2006, we selected 216 individual trees of 30 species to monitor, and this set expanded over time as new trees and fruit species were added. As individual trees died, they were dropped from the sample, and new trees of the same species were added to replace them. Because we expanded the number of species and trees per species under observation during the early years of phenology monitoring, the number of trees monitored in each month ranged from 207 to 413. In total, we analysed life-history data from 684 unique fruit-producing trees of 60 species consumed by capuchins and/or spider monkeys. The biographical data on fruit trees included the month when monitoring began, the month when monitoring ended, and the tree's status for each month of monitoring, which could be *alive* or *dead*. For trees that we determined to be *dead*, we defined the month of death as the month after the last visible sign of life that was recorded in the phenology records. Although we believe that these sampled trees capture the broad pattern of fruit tree mortality in SSR, the values should be interpreted with caution because trees that are newly added to the monitoring route to replace removed trees (e.g. after the death of a monitored tree) are not a truly random sample, but rather are selected because they are adult trees that appear capable of producing fruit.

### 2.6. Predictors of infant survival

We considered the possibility that each of the environmental predictors related to available ripe fruit biomass and climatic water balance could act over a range of different time scales [64]. We also considered the possibility that effects on survival could be a linear function of the environmental variable during the time window, or a ‘threshold function’ of the environmental variable such that its effects are only evident if conditions exceed a certain threshold during the time window [9]. Moreover, such effects might be immediate such that they increase the risk of death in the current month, or lagged as ecological effects ratchet through trophic levels to affect primate infant survival some time later.

We analysed infant survival on a monthly basis (see ‘Statistical analysis'). Because our one-month survival intervals were aligned to individual dates of birth rather than calendar months, the time windows were usually offset from the environmental predictors that were measured once per month. We used linear interpolation to expand the monthly fruit and SPEI data into daily time series. We then considered three distinct time windows at each time step of the survival models, each window ending on the date that closes the one-month interval during which survival was evaluated. The time windows began one month, six months and 12 months prior to this date. For each time window, we calculated mean daily fruit biomass over the time window, and the SPEI value on the closing date of the time window. Note that SPEI is already an integrative measure over a given time scale, and so taking the SPEI value on the date that closes each survival interval is analogous to the sliding window means used to aggregate the estimates of fruit biomass over the different time scales. Using the same set of time windows, we characterized each measured environmental variable as an extreme high (in top 10% of all values), an extreme low (bottom 10% of all values) or neither. In summary, there were six environmental variables at each time scale: mean ripe fruit biomass (continuous), extreme high fruit (true/false), extreme low fruit (true/false), SPEI value (continuous), extreme high SPEI (true/false) and extreme low SPEI (true/false) (electronic supplementary material, figures S1 and S2).

To account for the important influence of male infanticide on infant capuchin mortality at SSR, we calculated a binary variable of whether infanticide risk was present or absent during each survival interval. Following [52], we considered infanticide risk to be present if both of the following conditions were true*:* (i) an alpha male replacement occurred after the infant's estimated date of conception, which we define as 5.5 months prior to the infant's birth; and (ii) the infant was younger than 1 year old at the time that risk was evaluated. The estimated date of conception reflects the average gestation length of 5.5 months for SSR capuchins [65]. When these conditions are true, the young infant is unlikely to have been sired by the new alpha male, and would therefore be targeted under the sexual selection hypothesis of infanticide [66]. As it is not yet known whether male infanticide is a major source of infant mortality in SSR spider monkeys, and it is uncommon in other well-studied populations of spider monkeys [67], we did not consider infanticide risk in the spider monkey analysis.

### 2.7. Statistical analysis

We used time-varying mixed-effects Cox proportional hazards models to analyse the risk of dying over time as a function of the environmental predictors, separately for each species. This approach accounts for the hierarchical structure of our data: infants are naturally grouped by mother and birth cohort, which may result in correlated outcomes within grouping factors. All models included the grouping factors mother's identity and hydro-year as random effects or ‘shared frailty’ terms. We define a hydro-year as the 1-year period beginning on 15 May, the median start date of the rainy season in SSR. Thus, the 2015 hydro-year begins on 15 May 2015 and ends on 14 May 2016. The time-varying nature of these models indicates that each infant's outcome of interest (death or no death) was evaluated at multiple time steps—in our case, at one-month intervals beginning on the date of birth and ending at the minimum weaning age for each species—and that the environmental and social predictors were updated at each time step. We scaled the continuous environmental predictors to make their coefficients more easily interpretable. We used the R package coxme v. 2.2–10 [68] to fit the models in R v. 3.6.0 [69].

Because we were considering a large number of possible time windows and environmental predictors, we used an information theoretic approach, using Akaike's information criterion corrected for small sample size (AICc), to identify the most important influences on infant survival. For each primate species, we fit models of infant survival that included no environmental predictors (null models), models with each single environmental predictor, and models with all pairwise combinations of one fruit-related and one drought-related predictor (i.e. no more than one fruit-related or drought-related variable was entered in a given model). We systematically tested the models that were fit with two environmental variables for collinearity by calculating variance inflation factors (VIF) for each model. The maximum VIF value across all of these models was 1.174, and most values were close to 1, indicating no issues with collinearity. Furthermore, the absolute values of Pearson's correlation coefficients between the pairs of environmental variables included in the same model were all below 0.34 (electronic supplementary material, figures S3 and S4). To explore the possibility that some of the environmental variables could be conflated with the hydro-year random effect, we also fit models without hydro-year and found no qualitative differences in the results (results not shown). We included infanticide risk in all capuchin models (including the null model) because it is a known influence on the survival of infant capuchins.

After fitting the models, we carried out model averaging based on AICc to calculate model-averaged coefficients as well as 80%, 95% and 99% confidence intervals for each predictor of infant survival. Conditional model-averaged coefficients were calculated using the R package MuMIn v. 1.42.1 [70]. All models that were compared to each other using AICc were fit to the same dataset, as any rows with missing values were removed. Because the fruit biomass data began in 2008, they span a shorter time range than both the drought indices and the life-history data, and therefore the procedure described above excluded a considerable amount of infant survival data. In cases where fruit-related predictors did not predict infant survival, we refit the survival models using the entire set of life-history data by excluding all the fruit-related predictors.

To analyse the relationship between fruit tree mortality and climate variables, we fit binomial generalized linear mixed models (GLMMs) to the monthly tree death data, where the response variable was a two-column matrix counting the outcomes (death versus non-death) of monitored trees in the given month. As in the infant survival models, we included no more than one drought-related variable in each model, and we included random intercepts for tree species and hydro-year in all models. In addition, because we expected the effects of the climate variables on tree mortality to differ among the tree species, we included random slopes for tree species. We fit these models using the R package lme4 v. 1.1–21 [71].

## 3. Results

A total of 281 infant capuchins and 43 infant spider monkeys met the data requirements to be included in the survival analyses; these numbers were further restricted in some analyses depending on the availability of the environmental predictors. In both species, births occurred year-round but showed moderate seasonality: most spider monkey births occur during the dry season (December–May), whereas most capuchin births occur from late dry season to early wet season (April–July) (electronic supplementary material, figure S5).

**Figure 1.**
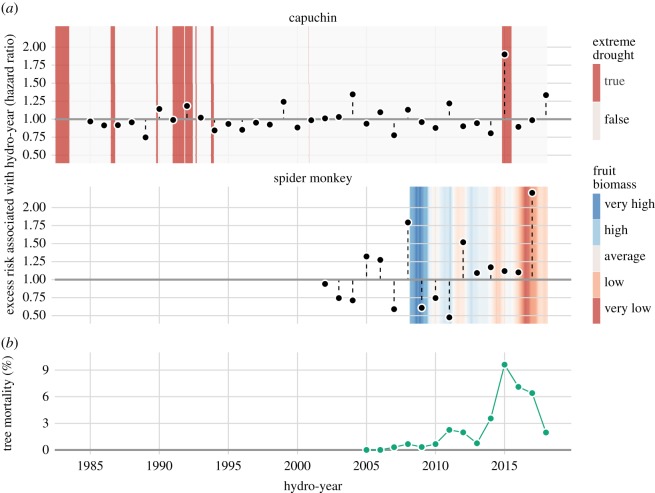
(*a*) Estimates of excess death hazard associated with individual hydro-years for infant capuchins (top) and spider monkeys (bottom); see text for model details. The shaded backgrounds show the environmental variables that were most strongly predictive of infant mortality in each species: presence of extreme drought at the 12-month time scale (capuchins) and mean of fruit biomass at the 12-month time scale (spider monkeys). The grey horizontal lines represent a hazard ratio of 1, corresponding to no difference from baseline mortality. (*b*) Annual mortality rates of fruit-producing trees monitored each hydro-year (*N* = 684 trees), calculated as the number of observed deaths in a given hydro-year divided by the total number of unique trees monitored in that hydro-year. The *x*-axis grid lines are positioned at the midpoints of the hydro-years (e.g. the 2015 tick mark indicates 15 November 2015).

Our decision to include infanticide risk in all capuchin models was strongly supported by the data: an infanticide-risk-only model that included both shared frailty terms (mother's identity and hydro-year) was supported by a ΔAICc value of 28.76 over an equivalent null model without infanticide risk, and the low-risk condition was associated with a hazard ratio (HR) of 0.25 (i.e. a 75% reduction in death hazard). The infanticide-risk-only model for capuchins also provides an opportunity to examine estimates for the different levels of the frailty terms (i.e. mother ID and hydro-year), after adjusting for risk status. This is informative for hydro-year, in particular, because it provides a picture of how much interannual variation in infant survival exists that is probably due to differences in environmental conditions before some of that variance is captured by the environmental predictors. In capuchins, the estimated hazard ratio associated with the hydro-year of extreme drought, 2015, was markedly higher than that of other years (figure 1*a*). Fruit tree mortality was also elevated during and in the 2 years following the 2015 drought (figure 1*b*), and available fruit biomass was exceptionally low during 2016 and 2017 (figure 1*a*; electronic supplementary material, figure S2). Importantly, these two co-occurring patterns following the drought—high fruit tree mortality and low available fruit biomass—were independently measured phenomena. Specifically, our estimates of available fruit biomass were derived from the phenology of *living trees only* (i.e. the abundance of each fruit tree species was assumed to be fixed throughout the study period). Because the abundances of many fruit-producing tree species were decreasing during this time of high mortality, we are probably overestimating fruit availability for these periods, and the effect of drought on fruit availability is probably more pronounced than we documented. Thirteen of the 31 infant capuchins that experienced part of the 2015/2016 drought, including eight of the nine infants born during the drought, did not survive despite low infanticide risk.

**Figure 2.**
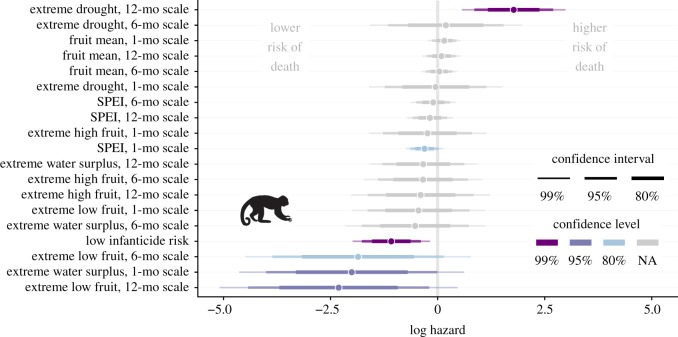
Model-averaged coefficients based on AICc for predictors of capuchin infant survival, with 80%, 95% and 99% confidence intervals. The confidence level, indicated by colour, represents the widest confidence interval that does not include zero.

**Figure 3.**
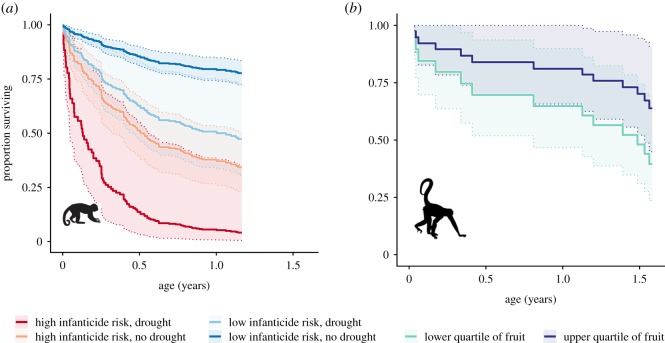
Predictions from the best-supported models of infant capuchin (*a*) and spider monkey (*b*) survival as a function of environmental covariates. The lines show predicted survival with 95% confidence bands for hypothetical infants that experience constant values of the indicated condition throughout infancy. In actual study animals, these values varied from month to month in our time-varying modelling framework.

In capuchins, the sample size for the initial model averaging procedure (which included fruit biomass variables) was 154 infants with 50 deaths during 1600 infant-months of observation. In this subset of data that was significantly reduced because of the later start date of the phenology records, the most informative predictors of capuchin infant survival were infanticide risk and the presence of extreme drought over a 12-month time scale (figure 2; electronic supplementary material, table S1). Some fruit-related variables were supported to a lesser degree, but their direction was contrary to our expectations (e.g. the presence of extreme low fruit was associated with reduced risk of death), and their wide confidence intervals suggests that the associations could be spurious (figure 2). We therefore fit new models of capuchin infant survival using the entire life-history dataset by excluding fruit-related variables. These models included 277 infants, with 87 deaths during 3011 infant-months of observation. Again, infanticide risk and the presence of extreme drought over a 12-month time scale were the best predictors of capuchin infant survival (ΔAICc = 2.26 compared to next-best model; ΔAICc = 8.29 compared to infanticide-risk-only model, electronic supplementary material, table S2). In the model that included only these two predictors and the frailty terms, reduced death hazard was associated with the low-risk infanticide condition (HR = 0.237), and increased death hazard was associated with drought (HR = 2.98) (figure 3).

In spider monkeys, exploration of the frailty terms in a null model revealed larger relative risks associated with mother than with hydro-year, and relatively large variance in both frailty terms. The estimate of the hazard for the year of severe drought in 2015 was not elevated relative to other years (figure 1*a*), probably due to the fact that none of the 11 reproductive-age spider monkey females who were in the group gave birth for the entire duration of the drought, from June 2015 to December 2016. This gap of 593 days without a birth was the longest such gap in the dataset by far: the next-largest gap lasted 334 days, and the median gap between successive births of spider monkeys was 61 days. The only two infant spider monkeys alive at the beginning of the drought died at about 1.5 years of age, one during the drought, and the other about six months after the drought ended.

**Figure 4.**
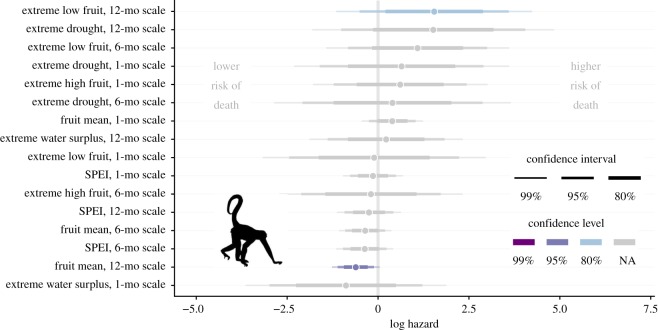
Model-averaged coefficients based on AICc for predictors of spider monkey infant survival. Note that two predictors (extreme high fruit, 12-month scale and extreme water surplus, six-month scale) were excluded because of model convergence problems. Plot elements are as described in figure 2.

For spider monkeys, the sample size for the initial model averaging procedure (including fruit biomass variables) was 27 infant spider monkeys with 13 deaths during 335 infant-months of observation. We excluded extreme high fruit on the 12-month scale and extreme water surplus on the six-month time scale because models that included these variables failed to converge. This occurred because none of the 13 spider monkey infant deaths occurred in the FALSE condition of these two binary variables. The only well-supported predictors of spider monkey infant survival were related to fruit availability, and the best predictor was mean fruit biomass in a 12-month time window (figure 4; electronic supplementary material, table S3). In the model that only included this variable, higher mean fruit biomass availability over the previous year was associated with reduced death hazard (HR = 0.543) (figure 3). Because no drought-related predictors were important predictors of infant survival in spider monkeys, we did not refit the survival models to include life-history data prior to the start of the fruit biomass data in 2008.

**Figure 5.**
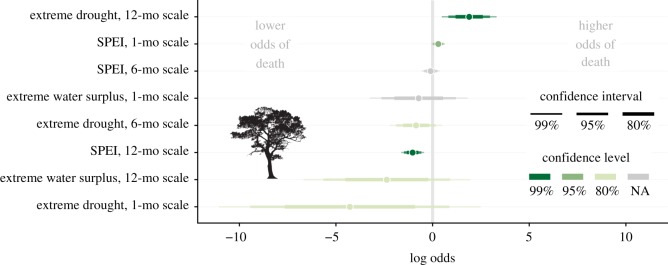
Coefficient plot of models predicting fruit tree mortality. Plot elements are as described in figure 2.

For the fruit trees, the dataset for the GLMMs included 684 distinct trees recorded over a period of 155 months, with a total of 150 deaths recorded during 52 248 tree-months of observation. We excluded one predictor variable, extreme water surplus on the six-month time scale because this model failed to converge as only one fruit tree died in the TRUE condition of this binary variable. The best climatic predictors of fruit tree mortality were measured at the relatively long 12-month time scale, and the continuous 12-month SPEI was strongly supported over all other models (ΔAICc = 25.8 compared to next-best model, figure 5; electronic supplementary material, table S4). Lower 12-month SPEI values, which represent water deficits (i.e. drought), predicted higher odds of fruit tree death.

## 4. Discussion

Our analysis of primate infant survival in relation to drought, ripe fruit biomass and social factors uncovered evidence that infant survival of both primate species varied in association with environmental conditions. However, we did not find evidence of shared responses to the same environmental variables in our two study-species. Rather, each species responded individualistically to different ecological constraints. In particular, capuchin infant survival was most sensitive to the presence of extreme drought, whereas spider monkey infant survival was most sensitive to the availability of ripe fruit. Because neither species is a strongly seasonal breeder, it is unlikely that differences in birth seasonality can explain the diverging patterns of infant mortality that we observed. Rather, these different sensitivities, in combination with the patterning of births during and after the drought, suggest the evolution of different strategies in capuchins and spider monkeys in response to extreme drought.

### 4.1. Environmental variability and primate infant mortality

Climatic water balance, as measured by SPEI, is an important determinant of the survival of infant capuchins. The presence of extreme drought predicted a threefold increase in the risk of death compared to non-drought conditions, after adjusting for infanticide risk. Interestingly, this effect became most salient in our models when the 12-month SPEI was converted to a binary variable indicating the presence or the absence of extreme drought over the preceding year. This pattern indicates that infant survival in capuchins may be buffered against typical interannual variability in climatic water balance, but that severe drought can exceed those tolerances and lead to rare, environmentally driven waves of high infant mortality. Extreme water surplus was not as strong a predictor of infant capuchin survival as extreme drought. This finding of sensitivity to extreme drought does not imply a seasonal difference in the relative risk of infant death. Although SSR experiences a long dry season each year, the SPEI is scaled within months, and therefore SPEI values are not consistently higher or lower during dry season months relative to wet season months. Indeed, the flora and fauna at SSR are seemingly well adapted to the periodic water shortages that occur each year during the extended dry season. In ecosystems such as SSR that experience long dry seasons, if a wet season fails to materialize—as it did during the 2015 hydro-year in association with a strong El Niño episode—two consecutive dry seasons can be linked together, leading to an 18-month period with little available surface water.

In spider monkeys, infant survival was more sensitive to the abundance of ripe fruit biomass than to drought-related variables. In interpreting these effects, it is important to consider how fruit availability could act to influence infant survival. During the first few months of life, when infant primates are most vulnerable, they are not consuming fruit. Infant spider monkeys begin foraging independently in the latter half of their first year of life, but may continue to nurse with declining frequency up to and sometimes beyond age two [72]. Hence, it is likely that low fruit availability could act on infant survival through two distinct pathways: (i) an indirect pathway in which the energetic status of the mother is reduced, which could then reduce the quality of infant care and the volume or nutritional content of milk; and (ii) a direct pathway in which older infants that have begun to forage independently are unable to obtain adequate food through their own inefficient foraging, and must therefore increase their reliance on the mother. Only the first pathway would be relevant to young infants, but either or both pathways could contribute to reduce survival of older unweaned infants. There were too few cases of infant deaths in spider monkeys to reliably distinguish between these hypotheses. In spider monkeys, low average ripe fruit availability during the preceding 12 months predicted lower infant survival. This observation is consistent with both scenarios described above and with previous findings from other ateline study systems [24]. It suggests that the loss of important fruit tree species—due to drought-associated tree mortality (figure 5), habitat disturbance, climate change or other processes—is likely to have particularly negative effects on spider monkey populations. However, this interpretation has an important caveat: the 12-month mean of available ripe fruit biomass shows a consistent long-term decline over time, despite some oscillations (electronic supplementary material, figure S2). Because of this long-term trend, if spider monkey infant mortality has increased over time due to other unrelated factors that we did not observe, then the association with average fruit biomass might be spurious. These possibilities would be difficult to disentangle because of the scarcity of data. In capuchins, we did not find sufficient evidence to conclude that ripe fruit biomass availability had a clear effect on infant survival.

### 4.2. Effects of severe drought

Taken together, our findings suggest differences in the vulnerability and adaptive responses of capuchins and spider monkeys to extreme drought. Although spider monkey infant survival was not strongly predicted by drought in our models, few spider monkey infants actually experienced the 2015 drought. It is therefore unrealistic to expect to see a strong signature of drought in spider monkey infant mortality. However, the long birth gap suggests that spider monkey adult females use a conservative bet-hedging strategy in which reproduction is inhibited (or spontaneously terminated) during periods of drought. Hence, while we are unable to conclude that infant mortality in spider monkeys is directly influenced by drought, we have evidence that their population dynamics are affected, nonetheless, through reduced offspring production. Further, the strikingly elevated fruit tree mortality rates during and after the drought suggests a lagged pathway connecting drought to reduced food availability, culminating in higher spider monkey infant mortality. Spider monkey responses to this drought are also consistent with those observed in other ateline populations in Brazil and Colombia, which show reduced birth rates and lagged population declines linked to unusually dry conditions associated with the El Niño Southern Oscillation [22]. These patterns are evidence of the vulnerability of spider monkeys to extreme climatic and ecological shifts. Their high degree of fission–fusion dynamics allows them to cope with shortage of food by fissioning into smaller subgroups during the lean periods [73,74], allowing spider monkeys to live in habitats where they could not live otherwise [75]. However, in SSR, it is likely that spider monkeys are routinely closer to their lower nutritional limits than capuchins because of their more highly selective diet and more energetically expensive locomotion [76]. Spider monkeys are absent from many seasonally dry forests of this region of Costa Rica, in part due to habitat fragmentation, historical hunting pressure and other anthropogenic factors [77]. This observation, and the lower densities of spider monkeys than capuchins at dry forest sites where both species persist [77], suggest that habitats like those at SSR are more marginal for spider monkeys than for capuchins. In such areas, spider monkeys may struggle more frequently to meet their energetic needs than capuchins. One possible explanation for this is that despite the substantial overlap in the fruit component of the diet, capuchins but not spider monkeys can fall back on invertebrates when fruit is scarce [41]. Therefore, spider monkeys may be more at risk of dying and experiencing nutrition-mediated disruptions in reproductive function as a result of prolonged fruit shortages than capuchins.

The exceptionally high rate of environment-linked mortality in infant capuchins, together with the observation that females continued to reproduce during the drought, indicates a response different to that of spider monkeys in which female capuchins continued to reproduce but were either incapable of keeping their dependent offspring alive or ceased investing in them in favour of future reproduction. During our regular censuses of capuchins, in the drought period we noted higher-than-usual rates of terminated pregnancies or stillbirths, inferred from obvious indicators of pregnancy (visibly distended ventral abdomen) followed in short succession by the absence of a live infant and a return to ‘normal’ body morphology, but we lack densely sampled hormonal data to track this definitively.

As long as a standing water source is available during the dry season at SSR, capuchins visit it almost every day and all group members descend to drink [78,79]. By contrast, spider monkeys rarely descend to the ground to drink water [79,80]. Although the reason for this behavioural difference is unclear, we speculate that spider monkeys can obtain nearly all of the water that they need during the dry season from their diet, whereas capuchins cannot. Differential dependence on sources of water in the diet offers a possible proximate mechanism to explain the immediate impact of extreme drought on capuchin infant mortality. Specifically, when drought causes long periods with no standing water, capuchins may experience physiological stress leading to greater mortality more rapidly than spider monkeys, which may only experience severe water stress when drought reduces fruit availability. Future studies could examine this hypothesis by assessing dehydration status via electrolyte balance or urinary specific gravity together with biomarkers of nutritional stress.

It is also possible that the strong relationship between capuchin infant mortality and drought is mediated through a food-related pathway that we were unable to quantify over time in this study: arthropod abundance. A major population crash of white-faced capuchins at Barro Colorado, Panama, has been attributed to a disruption in the abundance of edible arthropods during a period of fruit scarcity, which was triggered by extreme rainfall [17]. Although capuchins at SSR obtain most of their energy from fruit, arthropods contribute substantially to their diets in some months, and they represent the major source of protein in the capuchin diet throughout the year [63]. Previous research indicates that most arthropods are best viewed as high-quality fallback foods that supplement the fruit diet of capuchins at SSR [41]. However, outbreaks of caterpillars that typically occur in the early wet season (June to July) appear to be critical for capuchins to meet their energetic needs during this time [63], and caterpillar outbreaks may also represent an important resource for spider monkeys because, aside from these caterpillars, spider monkeys do not eat arthropods at other times of year. It is especially noteworthy that the early wet season is a time of low fruit abundance (electronic supplementary material, figure S6), and it also shortly follows the primary birth peak (electronic supplementary material, figure S5). If drought causes these caterpillars to fail to emerge, females with newborn infants may be unable to meet the energetic demands of lactation, leading to elevated infant mortality. Thus, an important area for future research in SSR will be to determine the relationship between drought and edible caterpillar abundance, and to determine the relative importance of caterpillars as a food source for the two species.

### 4.3. Primate mortality, conservation and climate change

Our findings indicate that extreme climate events—in particular, extreme drought and drought-associated changes in water, food availability or fruit tree mortality—can pose important risks for infant primates in seasonally dry tropical forests. Considerable uncertainty remains about how drought conditions are changing globally, but for the Neotropics in particular, a growing consensus points to greater drought intensity, length and frequency [13,14,81]. From a conservation perspective, such effects may be especially important for organisms in small populations because of the potential for local extinction. This risk is further compounded in organisms with intrinsically low reproductive rates because of their limited potential for rapid population growth. In this regard, non-human primates face a particularly worrisome future [29,82,83]. The specific mechanisms by which extreme climate events affect individual health and mortality risk in primate populations, potentially disrupting their population dynamics and threatening their status, should be a priority for future research.

## Ethics

The research was authorized by the Ministerio de Ambiente y Energía in Costa Rica. Data collection protocols were approved by the Life and Environmental Sciences Animal Care Committee of the University of Calgary (AC16-0255) and the Institutional and Animal Care and Use Committee of Tulane University (0399R2). The research complied with all the laws and guidelines of Costa Rica.

## Supplementary Material

Supplementary Tables S1 to S5

Reviewer comments

## Supplementary Material

Electronic Supplementary Material

## Data accessibility

Data and R code for survival models are available from the Dryad Digital Repository: https://doi.org/10.5061/dryad.6pt46j0 [84].
