# Supplementary material for: Differential impact of severe drought on infant mortality in two sympatric neotropical primates
Source: R Soc Open Sci. 2020 Apr 1;7(4):200302. doi: 10.1098/rsos.200302 (PMC7211846; doi:10.1098/rsos.200302)
Supplement: Electronic Supplementary Material [file rsos200302supp2.pdf]

Supplementary Materials for

Differential impact of severe drought on infant mortality in two sympatric neotropical primates

Fernando A. Campos, Urs Kalbitzer, Amanda D. Melin, Jeremy D. Hoga, Saul E. Cheves, Evin Murillo-Chacon, Adrián Guadamuz, Monica S. Myers, Colleen M. Schaffner, Katharine M. Jack, Filippo Aureli, Linda M. Fedigan

Supplementary Methods

Available ripe fruit biomass

We used 11 years (January 2008 to December 2018) of monthly phenological data on 552 individual trees of 46 fruit species (median of 8 trees per species per month) to estimate a ripe fruit availability index. The ripe fruit availability index expresses the relative amount of ripe fruit of a given species that is available in that month on scale from 0 (no ripe fruit on any monitored tree of that species) to 1 (all monitored trees of that species in maximum ripe-fruit phenophase and coverage). We then estimated the maximum ripe fruit productivity over the study area for each species using 423 100-m long botanical transects that were systematically arranged in a grid over the study area, covering a total area of 13.94 ha. We measured the diameter at breast height of all trees that were sufficiently large to bear fruit, and applied the formula from [1] to estimate the maximum ripe fruit biomass that each individual tree in the transect could produce based on the tree's diameter at breast height. Summing these individual estimates within-species and dividing by the total sampled area of the transects provides a measure of maximum ripe fruit

productivity (in kg/ha) for each species over the study area. We calculated monthly landscape estimates of available ripe fruit biomass for each species by multiplying these productivity estimates by the monthly availability indices. In effect, this measures what fraction of the potential maximum ripe fruit biomass is currently available.

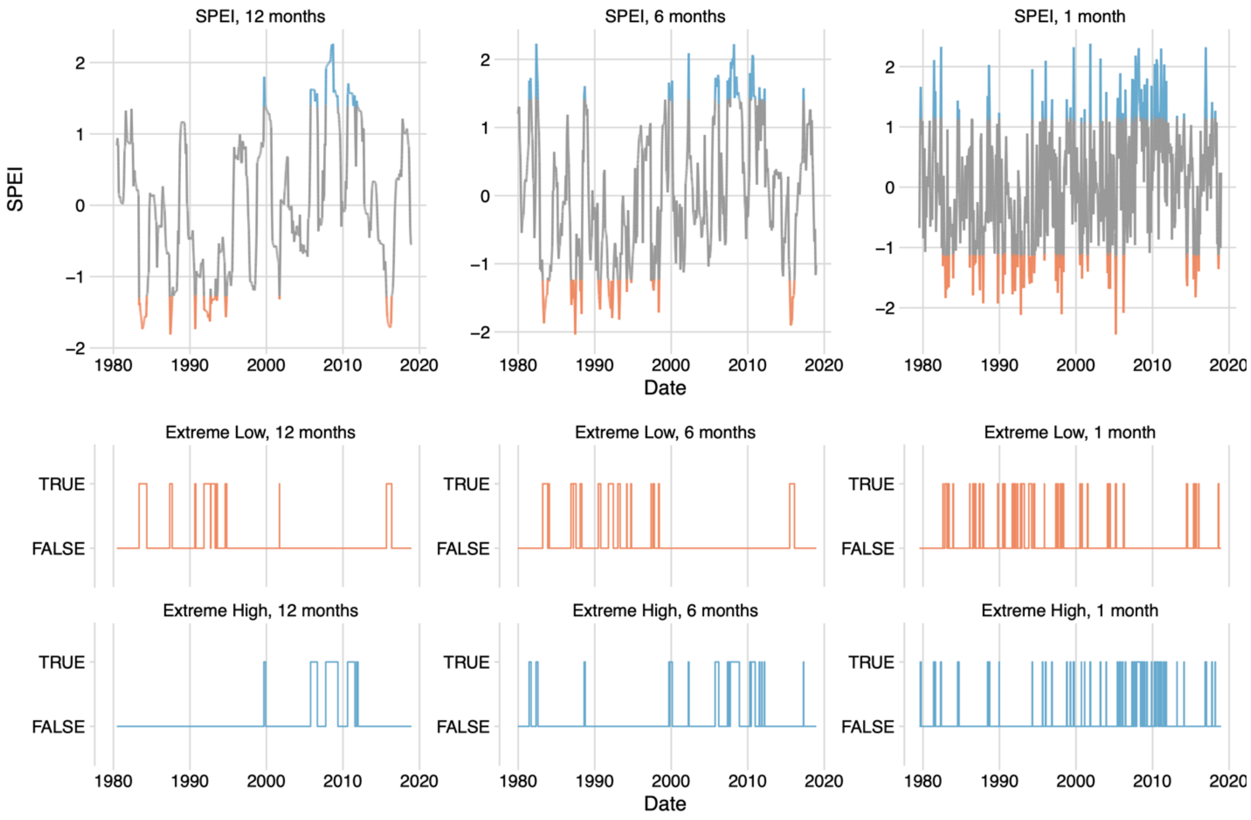

Figure S1. Drought-related variables used in the time-varying Cox models of infant survival at 12-month, 6-month, and 1-month time scales. The threshold-based variables ("Extreme Low" and "Extreme High") are true if the corresponding SPEI value is in the bottom 10% or top 90% of the distribution, respectively (shown by colour).

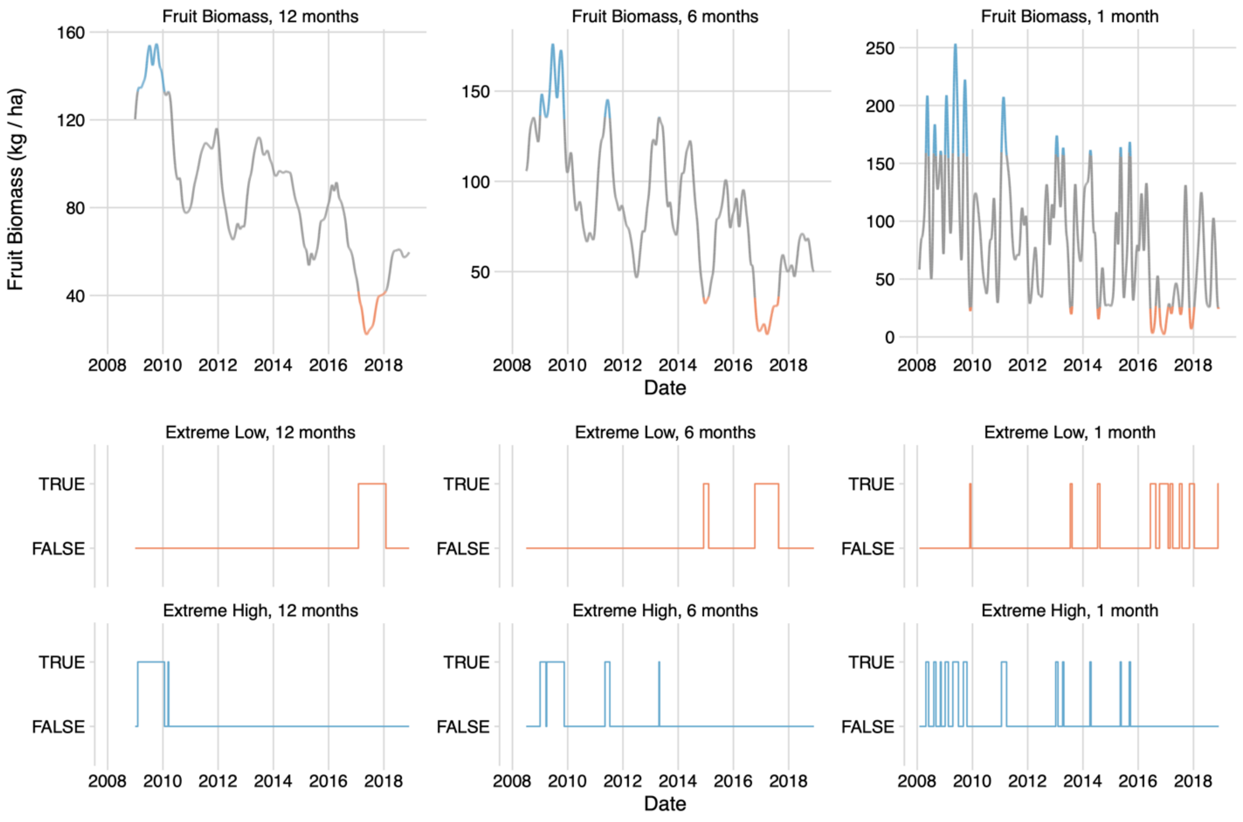

Figure S2. Variables related to available ripe-fruit biomass used in the time-varying Cox models of infant survival at 12-month, 6-month, and 1-month time scales. The threshold-based variables ("Extreme Low" and "Extreme High") are true if the corresponding mean fruit biomass value over the time window is in the bottom 10% or top 90% of the distribution, respectively (shown by colour).

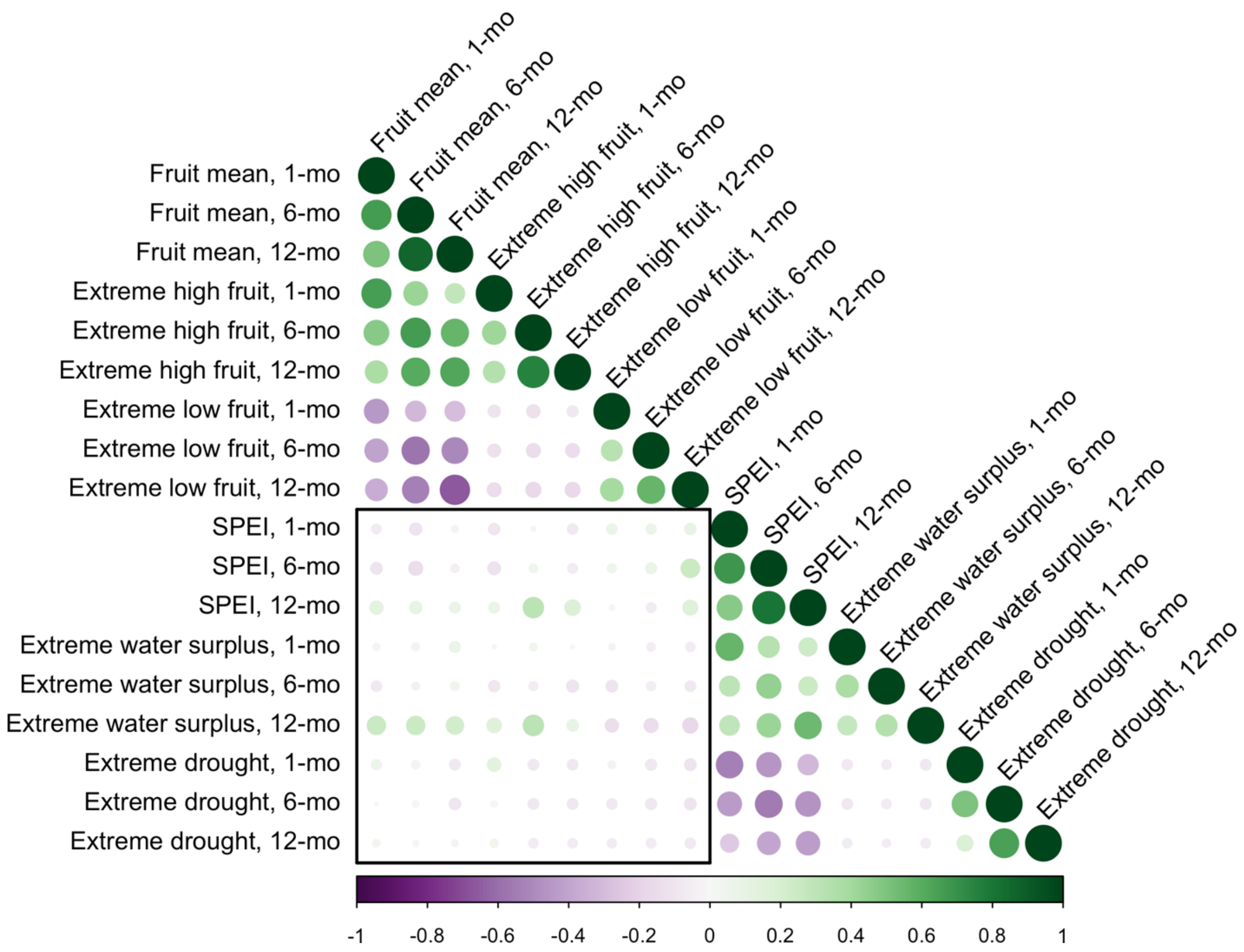

Figure S3. Correlation plot of fruit-related and drought-related environmental variables used to predict survival of infant capuchin monkeys. The size and colour of each dot indicate the Pearson correlation coefficient for the corresponding pair of variables. Pairwise variable combinations within the black box were included in our models, whereas all other variable combinations were excluded to avoid collinearity.

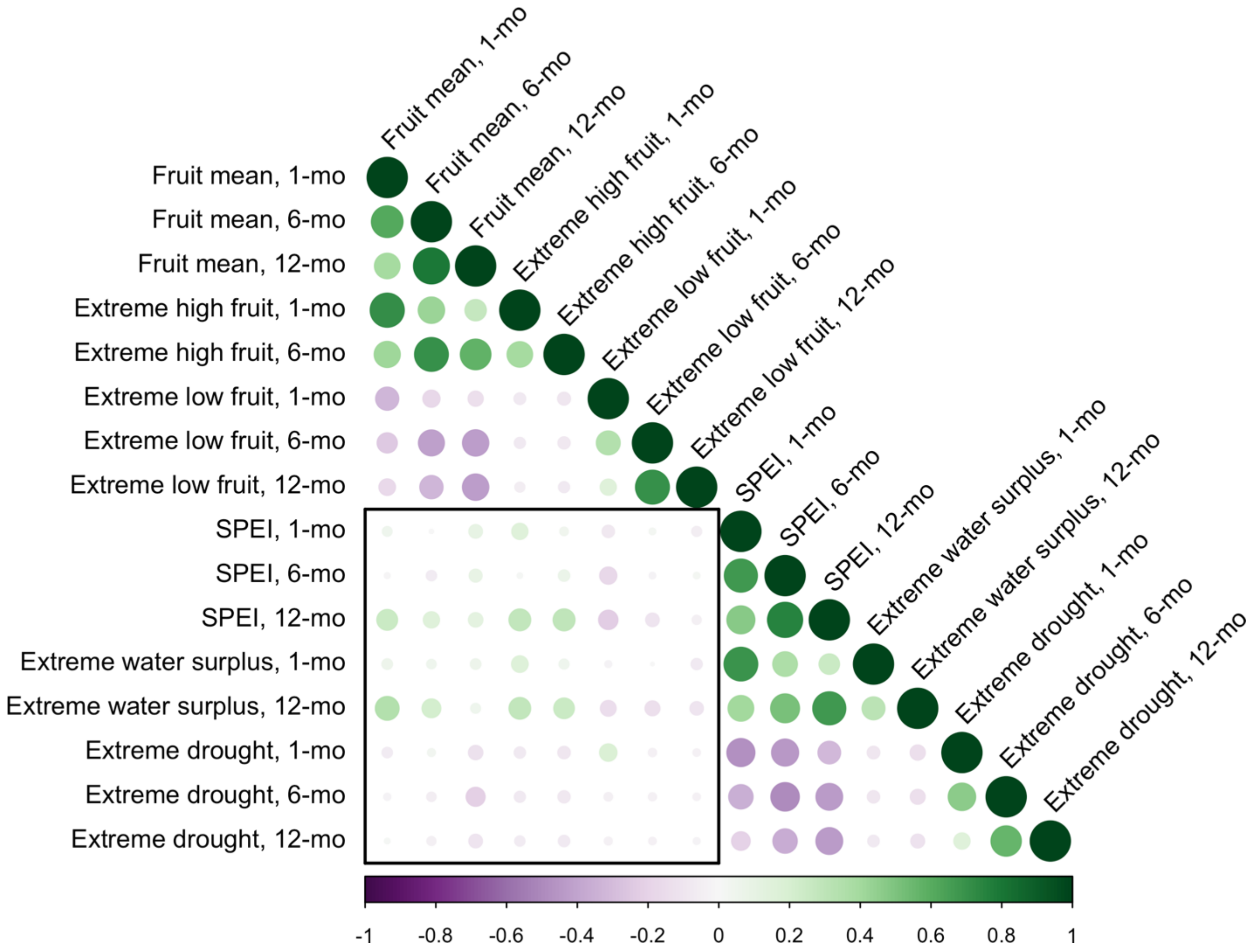

Figure S4. Correlation plot of fruit-related and drought-related environmental variables used to predict survival of infant spider monkeys. The size and colour of each dot indicate the Pearson correlation coefficient for the corresponding pair of variables. Pairwise variable combinations within the black box were included in our models, whereas all other variable combinations were excluded to avoid collinearity. Two additional variables—extreme high fruit on the 12-month scale and extreme water surplus on the 6-month scale—were excluded because of model convergence problems (see main text).

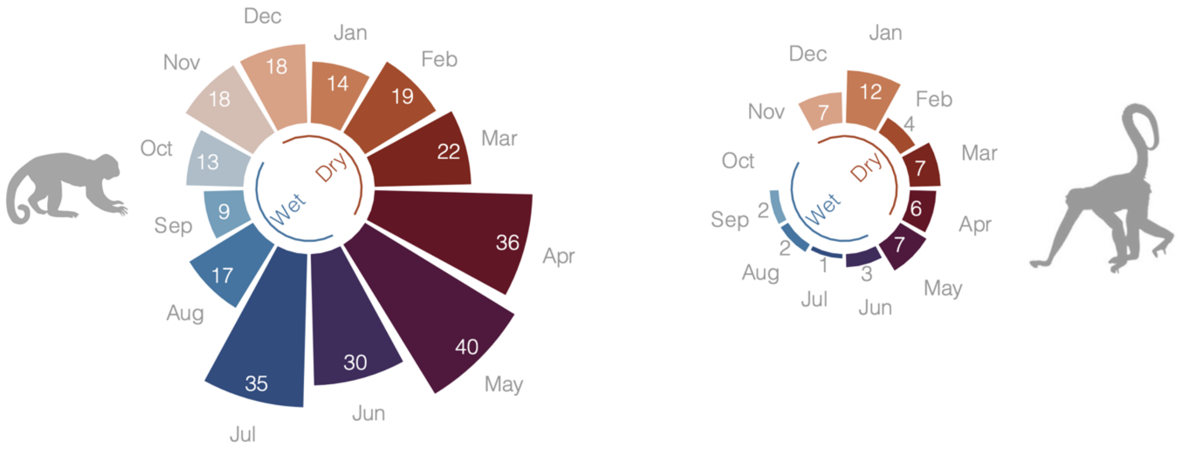

Figure S5. Circular histograms showing the seasonality of births in capuchins (left) and spider monkeys (right) in relation to wet and dry seasons. The numbers in each bar indicate the number of infant births that occurred in that month over the entire duration of the study.

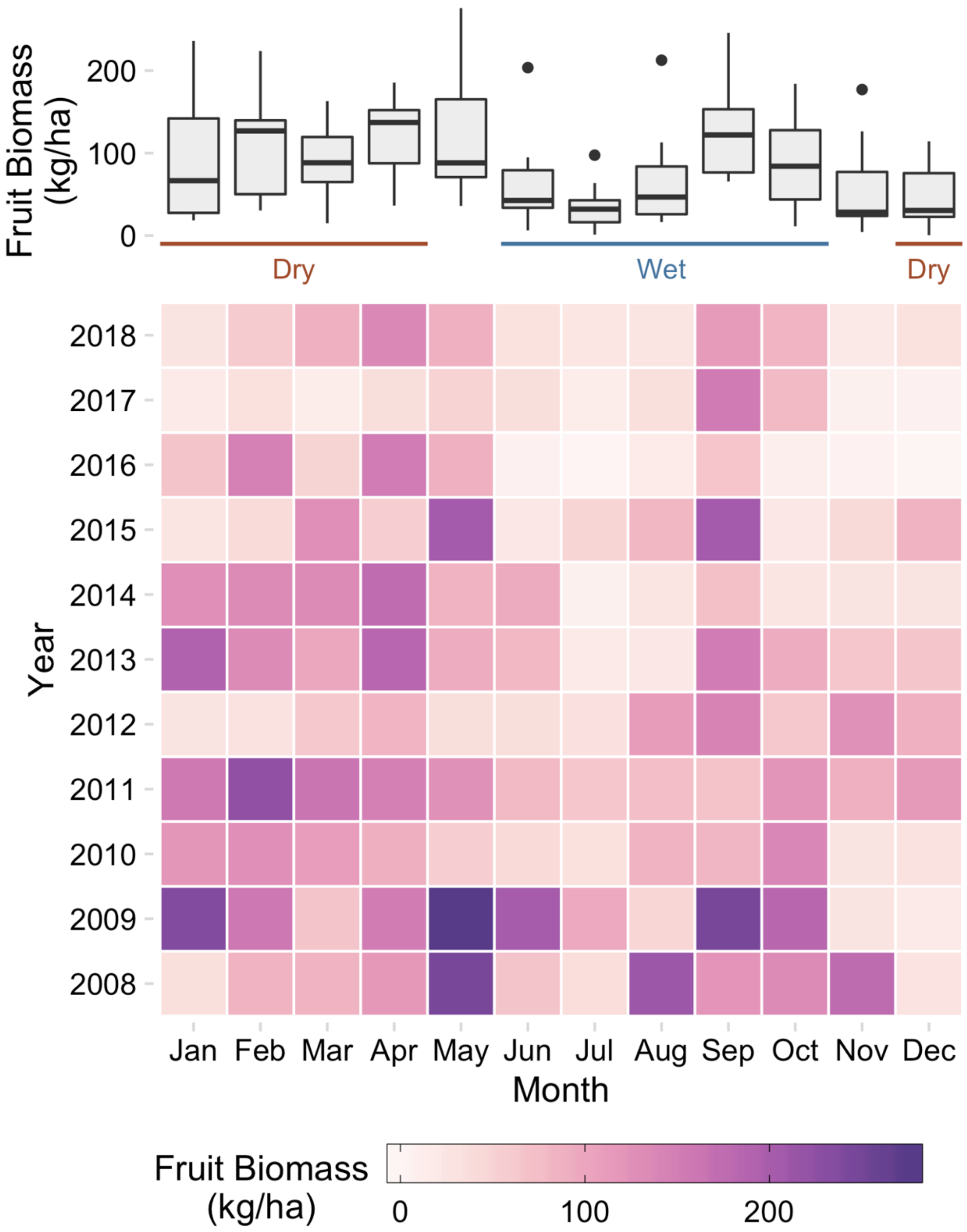

Figure S6. Monthly available fruit biomass over the study area, showing patterns of seasonality and inter-annual variability. The boxplots at the top for each month are based on the 11 grid cell values shown in the column below.

References

1. Peters RH, Cloutier S, Dubé D, Evans A, Hastings P, Kaiser H, Kohn D, Sarwer-Foner B. 1988 The allometry of the weight of fruit on trees and shrubs in Barbados. *Oecologia* **74**, 612–616. (doi:10.1007/BF00380061)
