# Supplementary material for: Differential impact of severe drought on infant mortality in two sympatric neotropical primates
Source: R Soc Open Sci. 2020 Apr 1;7(4):200302. doi: 10.1098/rsos.200302 (PMC7211846; doi:10.1098/rsos.200302)
Supplement: Reviewer comments [file rsos200302_review_history.pdf]

THE ROYAL SOCIETY
PUBLISHING

ROYAL SOCIETY
OPEN SCIENCE

Differential impact of severe drought on infant mortality in two sympatric neotropical primates

Fernando A. Campos, Urs Kalbitzer, Amanda D. Melin, Jeremy D. Hogan, Saul E. Cheves, Evin Murillo-Chacon, Adrián Guadamuz, Monica S. Myers, Colleen M. Schaffner, Katharine M. Jack, Filippo Aureli And Linda M. Fedigan

Article citation details
R. Soc. open sci. **7**: 200302.
http://dx.doi.org/10.1098/rsos.200302

Review timeline

Original submission:	24 February 2020
Final acceptance:	4 March 2020

Note: Reports are unedited and appear as submitted by the referee. The review history appears in chronological order.

Note: This manuscript was transferred from another Royal Society journal with peer review.

Review History

Decision letter (RSOS-200302.R0)

04-Mar-2020

Dear Dr Campos:

It is a pleasure to accept your manuscript entitled "Differential impact of severe drought on infant mortality in two sympatric neotropical primates" in its current form for publication in Royal Society Open Science. The comments of the reviewer(s) who reviewed your manuscript are included at the foot of this letter.

Please ensure that you send to the editorial office an editable version of your accepted manuscript, and individual files for each figure and table included in your manuscript. You can send these in a zip folder if more convenient. Failure to provide these files may delay the processing of your proof. You may disregard this request if you have already provided these files to the editorial office.

You can expect to receive a proof of your article in the near future. Please contact the editorial office (openscience_proofs@royalsociety.org) and the production office (openscience@royalsociety.org) to let us know if you are likely to be away from e-mail contact -- if

Reports © 2020 The Reviewers; Decision Letters © 2020 The Reviewers and Editors; Responses © 2020 The Reviewers, Editors and Authors. Published by the Royal Society under the terms of the Creative Commons Attribution License http://creativecommons.org/licenses/by/4.0/, which permits unrestricted use, provided the original author and source are credited

you are going to be away, please nominate a co-author (if available) to manage the proofing process, and ensure they are copied into your email to the journal.

Due to rapid publication and an extremely tight schedule, if comments are not received, your paper may experience a delay in publication.

Please see the Royal Society Publishing guidance on how you may share your accepted author manuscript at https://royalsociety.org/journals/ethics-policies/media-embargo/.

Thank you for your fine contribution. On behalf of the Editors of Royal Society Open Science, we look forward to your continued contributions to the Journal.

Kind regards,
Anita Kristiansen
Editorial Coordinator

Royal Society Open Science
openscience@royalsociety.org

on behalf of Dr Oliver Schülke (Associate Editor) and Professor Kevin Padian (Subject Editor).

Follow Royal Society Publishing on Twitter: @RSocPublishing
Follow Royal Society Publishing on Facebook:
https://www.facebook.com/RoyalSocietyPublishing.FanPage/
Read Royal Society Publishing's blog: https://blogs.royalsociety.org/publishing/
